# Editorial: Methods to develop and study animal cell degeneration disorders resembling human pathologies

**DOI:** 10.3389/fcell.2025.1575010

**Published:** 2025-04-04

**Authors:** Frank Edlich, Marlon R. Schneider

**Affiliations:** ^1^ Institute of Biochemistry, Faculty of Veterinary Medicine, University of Leipzig, Leipzig, Germany; ^2^ Institute of Veterinary Physiology, Faculty of Veterinary Medicine, University of Leipzig, Leipzig, Germany

**Keywords:** cell death programs, livestock animals, trauma, infection, cancer, metabolic disorders

Research on laboratory and domestic animals is a key element of current biomedical research. Animal models offer more insights into disease mechanisms than *in vitro* and *in silico* models, and can help bypass ethical and legal hurdles associated with human clinical trials. To be useful, an animal model must replicate the pathology of the human disease. Some animal models, especially rodent models, can fall short because they mimic only certain facets of a pathology. The reasons for the potentially low translational relevance of laboratory animals are manifold and include, beside sheer differences in size, histological, functional, and behavioral characteristics that are not shared with humans. Human activities alter the nutrition and living environments of many mammals, in particular those of domestic animals as dogs, cats, pigs, and cattle. It is therefore not surprising that humans and domestic or farm animals share a number of diseases, including those due to cell degeneration. Disorders like neurodegenerative diseases and cancers are becoming increasingly important to our domestic animals, and studying them can help to better understand human diseases. This Research Topic’s articles (3 original research papers, a perspective, and 3 review articles) delve into the different aspects of these fascinating translational connections. We anticipate that this Research Topic will help shape unifying concepts and clear the path for future progress.

Vogel et al. studied the fatty acid (FA) composition in the liver fat of German Holstein cows by comparing cows in their second lactation and those that had three or more lactations and examining differences in the periparturient phase, which ranges from 2 weeks before to 2 weeks after parturition. The most important results highlight significant differences in the FA C16:1n7 and polyunsaturated FA (PUFA) content in triacylglycerol (TAG) and free FAs. Therefore, the number of lactations influences the FA composition in the liver fat of cows. In particular, younger cows have a more favorable profile of beneficial FAs in the early postpartum period. These findings will aid understanding of dairy cows’ metabolic adaptations and inspire research into the role of fat metabolism in hepatocyte damage and liver disease.

Baalmann et al. studied the role of P2RY14, a UDP-glucose receptor, in kidney function. They focused on its effects on urine volume and sphingolipid metabolism in mice. P2ry14-deficient mice had significantly lower urine volumes compared to the wild type, which was not attributed to differences in urine osmolality and thus impaired water reabsorption. Quantitative analysis revealed changes in the number and structure of glomeruli in the area, indicating that P2RY14 is involved in maintaining kidney structure. Key differences in sphingolipid species suggests that P2RY14 influences kidney lipid metabolism. Overall, the results highlight the importance of P2RY14 for kidney health: it affects urine production, kidney structure, and sphingolipid metabolism.

Fietz et al. evaluated chronic inflammatory enteropathies in dogs, which may help us to understand the causes of and advance treatment strategies for human inflammatory bowel disease. In both dogs and humans, ultrastructural changes in intestinal epithelial cells impair barrier function, leading to increased intestinal permeability and inflammation. Future studies should examine how therapies, including dietary changes and probiotics, affect intestine structure and aid in mucosal repair.

Wang et al. highlight the value of ductal ligation models for understanding the damage and regeneration mechanisms of salivary glands. Ductal ligation leads to atrophy of the salivary glands, which is characterized by the apoptosis of acinar cells and reduced proliferation of myoepithelial cells. Long-term ligatures show pronounced fibrosis and a drastic reduction in salivary secretion. Mitochondrial apoptosis appears to exert a significant influence and has been identified as an underlying cause in diseases such as the autoimmune illness Sjögren’s syndrome. The results of these studies could offer new therapeutic and/or regenerative approaches for salivary gland diseases.

In their comprehensive review of osteoarthritis (OA) in small animals, Theyse and Mazur emphasize the impact of this debilitating disease. OA is characterized by degradation of the articular cartilage, inflammation of the synovial membrane, and changes in the subchondral bone. These changes cause pain, hinder movement, and reduce the quality of life of affected animals. Adipokines derived from adipose tissue, such as leptin, influence inflammation and cartilage metabolism. Dogs and cats can serve as valuable models for the study of obesity and OA, particularly with regard to the implications of adipokine interactions. This approach could lead to more effective treatments and improve our understanding of disease mechanisms.

Complementarily, Jia et al. explored the protective role of irisin, a myokine induced by exercise, against chondrocyte pyroptosis in OA, using a rat model. The study identified genes that are differentially expressed in response to exercise in Sprague-Dawley rats using the GSE74898 dataset. The authors identified a total of 389 overlapping genes, suggesting modulation of inflammatory responses in chondrocytes by exercise. Significant downregulation of irisin levels was observed in the damaged areas of articular cartilage in patients with knee OA. Irisin could therefore play a protective role in maintaining cartilage integrity. In rats that underwent treadmill training, knee joint inflammation and cell death markers decreased, while histological values improved. The exercise program increased irisin expression, which was linked to a decrease in pyroptosis of chondrocytes. The study points out that physical activity plays a vital role in treating OA.

Focusing on reptile-associated salmonellosis (RAS), Pees et al. summarize the prevalence of *Salmonella* in reptiles and how it affects human health. A total of 138 data sets, covering over 12,500 reptiles, were obtained from 77 publications and analyzed. *Salmonella* is widespread in reptiles, with a significant detection rate in cloacal swabs and fecal samples. The most frequently identified serovars belong to subspecies I, followed by subspecies IIIb and II. A total of 3,025 cases of RAS in humans have been reported, most of which were due to contact with reptiles. Young children appear to be particularly susceptible to RAS, highlighting the need for awareness and prevention measures.

Häcker also provides a comprehensive overview of chlamydial infections in pigs, focusing on *Chlamydia suis, Chlamydia abortus,* and *Chlamydia pecorum*. These species have zoonotic potential, posing a direct threat to human health. The existing literature highlights the crucial role of the type III secretion system in their virulence. It enables them to manipulate the functions of host cells. *Chlamydia suis* is the most common type of *chlamydia* in pigs, causing reproductive problems like abortions and infertility with considerable impact on pig production. *Chlamydia abortus*, which causes abortions in sheep, can also infect pigs. *Chlamydia pecorum* is associated with respiratory and reproductive disorders. It is often difficult to identify the clinical signs of these infections. However, early diagnosis and treatment are crucial. The author emphasizes that knowing the immunogenic properties of chlamydial proteins is key to developing vaccine candidates. Understanding chlamydial infections in pigs better, including how they spread and the immune system’s response, will improve animal health and reduce the risk of transmission to humans.

